# A 12-year trend analysis of the incidence of gastrointestinal cancers in East Azerbaijan: last updated results of an ongoing population-based cancer registry

**DOI:** 10.1186/s12885-019-6008-3

**Published:** 2019-08-07

**Authors:** Mohammad Hossein Somi, Roya Dolatkhah, Sepideh Sepahi, Mina Belalzadeh, Shahnaz Naghashi, Mohammad Asghari Jafarabadi

**Affiliations:** 1 Liver and Gastrointestinal Diseases Research Center, Tabriz University of Medical Sciences, Tabriz, Iran; 2 Hematology and Oncology Research Center, Tabriz University of Medical Sciences, Tabriz, Iran; 3 Cancer Registry Office, Tabriz University of Medical Sciences, Tabriz, Iran; 4 Road Traffic Injury Research Center, Tabriz University of Medical Sciences, Tabriz, Iran

**Keywords:** Trend, Incidence, Cancer, East Azerbaijan, Gastrointestinal

## Abstract

**Background:**

The most recent results of Global Cancer Statistics indicated that gastrointestinal cancers, including gastric, colorectal, esophageal, and liver cancers, are among the most commonly diagnosed cancers worldwide. Previous reports from cancer registries in East Azerbaijan have shown that there is a high incidence of gastrointestinal cancer in this region, so we performed a trend analysis to determine the pattern of change over the last decade.

**Methods:**

In total, 12 years of cancer registry data were collected from different sources in East Azerbaijan, and a data quality check was performed to ensure clean data. Using the 2000 World Health Organization standard population, we then generated age-standardized incidence rates (ASRs) for different cancers, and for each year from 1383 to 1394 of the Persian calendar (i.e., 19 March 2004 to 20 March 2015). Annual percent changes (APCs) and Average annual percent changes (AAPCs) in the ASRs for esophageal, gastric, small intestine, colorectal, anal, liver, gallbladder, and pancreatic cancers were calculated using Joinpoint Software (Version 4.5.0.1, June 2017).

**Results:**

An increase in most types of cancer was observed during the study period. The ASR for colorectal cancer increased from 2.9 to 13.6 per 100,000 women (APC, 9.7%) and from 2.2 to 17.8 per 100,000 men (APC, 10.2%). The ASR for gastric cancer showed a slight increasing trend from 10.5 to 13.5 per 100,000 women (APC, 1.3%) and from 3.1 to 29.9 per 100,000 men (APC, 3.2%). However, trend analysis showed a decreasing pattern for the ASR of esophageal cancer in both genders (APC,− 3%), with APCs of − 1.1% in females and − 0.4% in males.

**Conclusions:**

The latest results of the East Azerbaijan Population-Based Cancer Registry indicate that gastrointestinal cancers remain common, with significant increasing trends in their ASRs. Improved screening and early detection are needed in this region.

## Background

The most recent results from Global Cancer Statistics 2018 showed that gastrointestinal cancers, including colorectal, stomach, and liver cancers, represent the most common causes of cancer death worldwide [1]. Although the highest incidences of colorectal cancer are generally seen in high-income countries, liver, gastric, and esophageal cancers are more common in low-income countries. Unfortunately, the significantly poorer prognosis of these cancers means that cancer deaths are more common in less economically developed countries [1, 2]. To understand the burden and monitor trends in cancer rates, a national pathology-based cancer registry program was introduced throughout Iran and East Azerbaijan in 2001. The first estimates of the incidence of cancer in East Azerbaijan were reported for the period from March 2006 to March 2007, revealing age-standardized rates (ASRs) of 164.3 per 100,000 men and 130.9 per 100,000 women [3]. In both sexes, gastrointestinal cancers were among the top five most common cancers. The latest updated results from Eat Azerbaijan showed that gastric cancer was the most common cancer, by an ASR of 23.1 and 7.7 for males and females respectively. Also the ASRs for esophageal and colorectal cancers were 9.69 and 11.2 in males and 7.35 and 8.93 in females [4]. According to last National cancer registry reports of Iran, north and northwest areas of Iran had the highest incidence and mortality of GI cancers [5]. Located in the northwest of Iran, East Azerbaijan is the largest and most populated province, and has a high incidence of gastrointestinal tract cancers, including esophagus and stomach cancers [6, 7]. Higher prevalence of some well-known risk factors including *H. pylori* infection, smoking, opium use, higher usage of biomass and so exposure to polycyclic aromatic hydrocarbons (PAH), and dietary factors may explain higher incidence of GI cancers in North/Northwest of Iran [7].

In this study, we aimed to analyze and summarize the data from our cancer registry over a 12 years period to show the trend in the incidence of gastrointestinal cancers in East Azerbaijan.

## Methods

The cancer registry data were collected from different sources in East Azerbaijan from March 19, 2004, to March 20, 2015. The East Azerbaijan cancer registry program started as a pathology-based cancer registry, aiming to cover all histological and pathological reports of the province. The first population-based cancer registry started in March 2006, after which efforts were made in subsequent years to enhance the coverage of registered data. The population-based cancer registry program was completed in 2015 and we expect the results of this to be published soon. This registry sources data from pathology laboratories, hospital medical records, and cause of death registries, as well as radiotherapy, hematology, and imaging departments, to estimate the rates of different cancers in East Azerbaijan Province [6].

After collecting the data, a final quality check was performed to achieve clean data, identify duplicates, and reconcile new data in comparison with previous data. The following information was mandatory for case inclusion in the study: first and last name, birth date, fathers’ name, sex, place of residence, and date of diagnosis, as well as the morphology (i.e., histology, behavior, and grade) and topography (primary site of origin) of the tumor based on the International Classification of Diseases for Oncology, Third Edition (ICD-O-3) [8]. Using the World Health Organization standard population for 2000 [9], ASRs were generated for different cancers for each year of the Persian calendar, from 1383 to 1394 (i.e., March 19, 2004, to March 20, 2015).

We used Joinpoint Trend Analysis Software (Version 4.5.1.0) for regression analysis, which provided a useful way to summarize the observed trends in cancer and to identify significant changes in ASRs [10]. Connecting linear segments on a log scale allowed us to characterize the annual percent changes (APCs) and average annual percent changes (AAPCs) of the ASRs for esophageal, gastric, small intestinal, colorectal, anal, liver, gallbladder, and pancreatic cancers [10]. All diagnoses were based on pathology reports and/or clinical information. The program uses a sequence of permutation tests to select the final model, so we considered a maximum of two Joinpoints between the three time periods and tests of significance were conducted using the Monte Carlo permutation method [10].

The ethics committee of Tabriz University of Medical Sciences has been approved this project. As the ethics rules of EA-PBCR, all patients’ information and records were stored confidentially [IRB code: IR.TBZMED.REC.1395.18]. Consent to participate was not applicable.

## Results

During the period of interest, 15,614 cases of gastrointestinal cancer were registered. Of these, 3537 were esophageal (22.7%), 6162 were gastric (39.7%), 4331 were colorectal (27.7%), 555 were liver (3.6%), 421 were small intestinal (2.7%), 105 were anal (0.7%), 253 were gallbladder (1.6%), and 250 were pancreatic (1.6%).

For esophageal cancer, the overall APC was − 3%, and this decline in ASR was statistically significant (*P* ≤ 0.05). For gastric cancer, we observed an increasing APC of 1.8% (P ≤ 0.05), which indicates the ASR increased from 7.9 to 21.2 per 100,000 people. The overall APC for colorectal cancer was 7.6%, but was not significant statistically (*P* = 0.13), reflecting an ASR increase from 3.0 to 15.6 per 100,000 people from 2004 to 2015. For liver cancer, the overall APC was 17.2% (*P* ≤ 0.05) and was larger in men (APC, 21.3%). The ASR increased from 0.9 to 4.9 per 100,000 people (Tables 1, 2). The AAPCs were the same as the APCs in all cancer trend results.

**Table 1:** Annual Percent Changes of Gastrointestinal Cancers in East Azerbaijan, from 2004 to 2015

Cancer Site	Overall 12 years (2004–2015)				Segment 1			Segment 2			Segment 3		
	APC (%)	Lower CI	Upper CI	P Value	APC (%)	Lower CI	Upper CI	APC (%)	Lower CI	Upper CI	APC (%)	Lower CI	Upper CI
Esophagus													
Both	-3	−6.1	0.2	0.05	5.9	−6.7	20.3	−16.2	−51.3	44.2	4.5	−21.3	38.7
Male	−0.4	−3.8	3.0	0.19	6.8	−12.3	30.2	−8.5	−40.2	40.0	8.6	−51.0	140.4
Female	−1.1	−3.6	1.5	0.10	0.9	−5.9	8.2	−11.0	−44.1	41.6	20.1	−21.2	83.3
Stomach													
Both	1.8	−3.4	7.2	0.01	29.5	−24.7	122.7	−9.4	−26.2	11.2	36.1	−37.0	194.0
Male	3.2	−3.0	9.7	0.17	38.0	−50.3	283.7	−7.4	−30.9	24.0	34.8	−56.7	320.3
Female	1.3	−3.4	6.2	0.00	−11.1	−45.5	44.8	−3.7	−12.5	6.0	45.1	−8.0	128.9
Colorectal													
Both	7.6	2.7	12.7	0.13	23.4^a^	1.8	49.5	−9.6	−49.4	61.7	16.8	−10.2	51.8
Male	10.2^a^	5.5	15.1	0.11	46.6	−22.7	177.9	0.7	−22.1	30.1	18.4	−14.8	64.6
Female	9.7^a^	5.7	13.8	0.19	18.7^a^	6.3	32.6	−3.4	−41.7	60.1	18.6	−21.1	78.4
Anus													
Both	−0.2	−8.7	9.0	0.14	13.7^a^	0.2	29.0	−34.2	−68.8	39.1	57.9	−21.6	218.2
Male	0.9	−10.8	14.1	0.38	17.5	−9.1	51.9	−35.8	−90.1	317.5	55.1	− 73.9	821.3
Female	4.3	−1.3	10.2	0.02	45.4	−43.7	275.5	−1.9	−13.6	11.4	20.6	−38.5	136.7
Gallbladder													
Both	3.5	−7.1	15.2	0.00	22.6^a^	9.6	37.1	−63.0	−89.1	26.0	266.1^a^	8.9	1130.8
Male	6.0	−4.2	17.3	0.07	13.5	−4.0	34.2	−55.3	−92.0	149.7	269.7	−62.4	3534.0
Female	–	–	–	–	–	–	–	–	–	–	–	–	–
Liver													
Both	17.2	6.2	29.3	0.00	−6.4	−40.5	47.3	−1.2	−8.8	7.0	138.2^a^	76.9	220.7
Male	21.3^a^	9.4	34.4	0.00	−14.5	−54.6	60.8	4.4	−6.4	16.5	139.9^a^	62.4	254.2
Female	16.3^a^	5.6	28.0	0.02	−1.1	−14.1	13.9	−6.9	−56.8	100.7	148.0^a^	45.6	322.7
Pancreas													
Both	28.6^a^	15.2	43.6	0.02	−3.3	−45.6	72.2	22.9	−49.6	199.8	98.7	−24.2	421.2
Male	33.5^a^	17.8	51.3	0.05	2.3	−43.4	84.9	38.4	−84.9	1167.2	98.3	−48.3	659.7
Female	28.5^a^	17.2	41.0	0.02	−14.2	− 78.6	244.2	21.3	−16.8	76.9	90.8	−12.1	314.1
Small Intestine													
Both	7.6^a^	1.3	14.2	0.12	99.8	−68.9	1183.8	−1.3	−15.7	15.5	34.6	−34.4	176.4
Male	9.3^a^	2.0	17.2	0.36	66.0	−91.4	3108.9	−0.1	−20.2	25.0	45.7	−47.2	302.0
Female	9.9^a^	3.3	16.8	0.12	68.8	−39.1	368.0	−4.0	−53.1	96.4	14.7	−16.4	57.2

^a^Indicate that the Annual Percent Change (APC) is significantly different from zero at alpha = 0.05

**Table 2:** Age Standardized Incidence Rates of Gastrointestinal Cancers in East Azerbaijan, from 2004 to 2015

Cancer Site	Esophagus									Stomach									Colorectal									Liver								
	Both			Male			Female			Both			Male			Female			Both			Male			Female			Both			Male			Female		
Year	Obs.ASR	SE	Mod.ASR	Ob.ASR	SE	Mod.ASR	Obs.ASR	SE	Mod.ASR	Obs.ASR	SE	Mod.ASR	Obs.ASR	SE	Mod.ASR	Obs.ASR	SE	Mod.ASR	Obs.ASR	SE	Mod.ASR	Obs.ASR	SE	Mod.ASR	Obs.ASR	SE	Mod.ASR	Obs.ASR	SE	Mod.ASR	Obs.ASR	SE	Mod.ASR	Obs.ASR	SE	Mod. ASR
2004	7.0	0.5	10.4	5.1	0.6	8.7	6.9	0.6	7.9	7.9	0.5	9.3	3.1	0.4	16.8	10.5	0.8	9.6	3.0	0.3	6.9	2.2	0.4	5.9	2.9	0.4	5.1	0.9	0.2	1.0	0.9	0.2	0.9	0.7	0.2	0.8
2005	10.9	0.6	10.1	9.9	0.8	8.7	8.7	0.7	7.8	14.7	0.7	12.0	18.6	1.1	17.3	8.7	0.7	9.1	7.0	0.5	7.4	6.2	0.6	6.5	5.6	0.6	5.6	1.2	0.2	1.0	1.2	0.3	0.9	0.9	0.2	0.8
2006	9.6	0.6	9.8	8.6	0.7	8.7	7.6	0.7	7.7	13.7	0.7	15.6	16.6	1.0	17.9	6.7	0.6	8.6	7.3	0.5	8.0	6.9	0.6	7.1	5.6	0.6	6.1	0.7	0.2	0.9	0.6	0.2	0.9	0.7	0.2	0.8
2007	9.5	0.6	9.5	8.9	0.7	8.6	6.9	0.6	7.6	19.2	0.8	20.2	24.0	1.2	18.4	8.7	0.7	8.2	10.4	0.6	8.6	10.2	0.8	7.9	7.2	0.6	6.7	0.9	0.2	0.9	0.7	0.2	0.9	0.6	0.2	0.7
2008	10.3	0.6	9.2	8.9	0.7	8.6	8.4	0.7	7.5	15.8	0.7	18.3	19.4	1.1	19.0	7.3	0.6	7.8	10.2	0.6	9.3	9.7	0.8	8.7	7.5	0.7	7.4	0.7	0.2	0.9	0.9	0.2	0.9	0.8	0.2	0.7
2009	11.0	0.6	8.9	10.1	0.8	8.6	8.0	0.7	7.4	18.0	0.8	16.6	22.6	1.1	19.6	7.7	0.7	7.4	10.9	0.6	1.0	9.8	0.7	9.5	8.5	0.7	8.1	0.9	0.2	0.9	1.0	0.2	0.9	0.5	0.2	0.7
2010	10.6	0.6	8.6	9.1	0.7	8.5	8.5	0.7	7.4	19.7	0.8	15.0	24.1	1.2	20.3	8.8	0.7	7.0	15.6	0.7	10.7	13.6	0.9	10.5	12.3	0.9	8.9	0.9	0.2	0.9	0.7	0.2	0.9	0.7	0.2	0.7
2011	7.8	0.5	8.4	9.7	0.7	8.5	6.0	0.6	7.3	13.1	0.6	13.6	19.7	1.1	20.9	6.1	0.6	6.7	10.2	0.5	11.5	9.7	0.7	11.6	10.4	0.8	9.7	0.9	0.2	0.8	1.0	0.3	0.9	0.8	0.2	0.7
2012	6.6	0.5	8.1	6.8	0.6	8.4	6.2	0.6	7.2	10.5	0.6	12.3	14.9	0.9	21.6	5.8	0.6	6.3	9.3	0.5	12.4	10.1	0.8	12.8	8.1	0.7	10.7	1.2	0.2	0.8	1.3	0.3	1.0	1.0	0.3	0.6
2013	6.6	0.4	7.9	6.9	0.6	8.4	6.2	0.6	7.1	11.2	0.6	11.2	15.8	1.0	22.3	6.3	0.6	6.0	12.0	0.6	13.4	12.8	0.9	14.1	10.9	0.8	11.7	0.8	0.2	0.8	1.0	0.3	1.0	0.5	0.2	0.6
2014	7.6	0.5	7.7	8.7	0.7	8.4	6.3	0.6	7.0	13.9	0.64	15.2	19.0	1.0	23.0	8.3	0.7	8.9	14.3	0.7	14.4	15.4	0.9	15.5	12.8	0.9	12.9	1.3	0.2	1.9	1.6	0.3	2.4	1.0	0.2	1.5
2015	7.6	0.4	7.4	8.0	0.7	8.3	8.3	0.7	7.0	21.2	0.8	20.7	29.9	1.3	23.7	13.5	0.8	13.2	15.6	0.6	15.5	17.8	1.0	17.1	13.6	0.8	14.1	4.9	0.4	4.7	6.2	0.6	5.9	3.7	0.4	3.6

*Obs.ASR* Observed Age Standardized Incidence Rate, *SE* Standard Error, *Mod.ASR* Modeled Age Standardized Incidence Rate

### Esophageal cancer

**Fig. 1:**
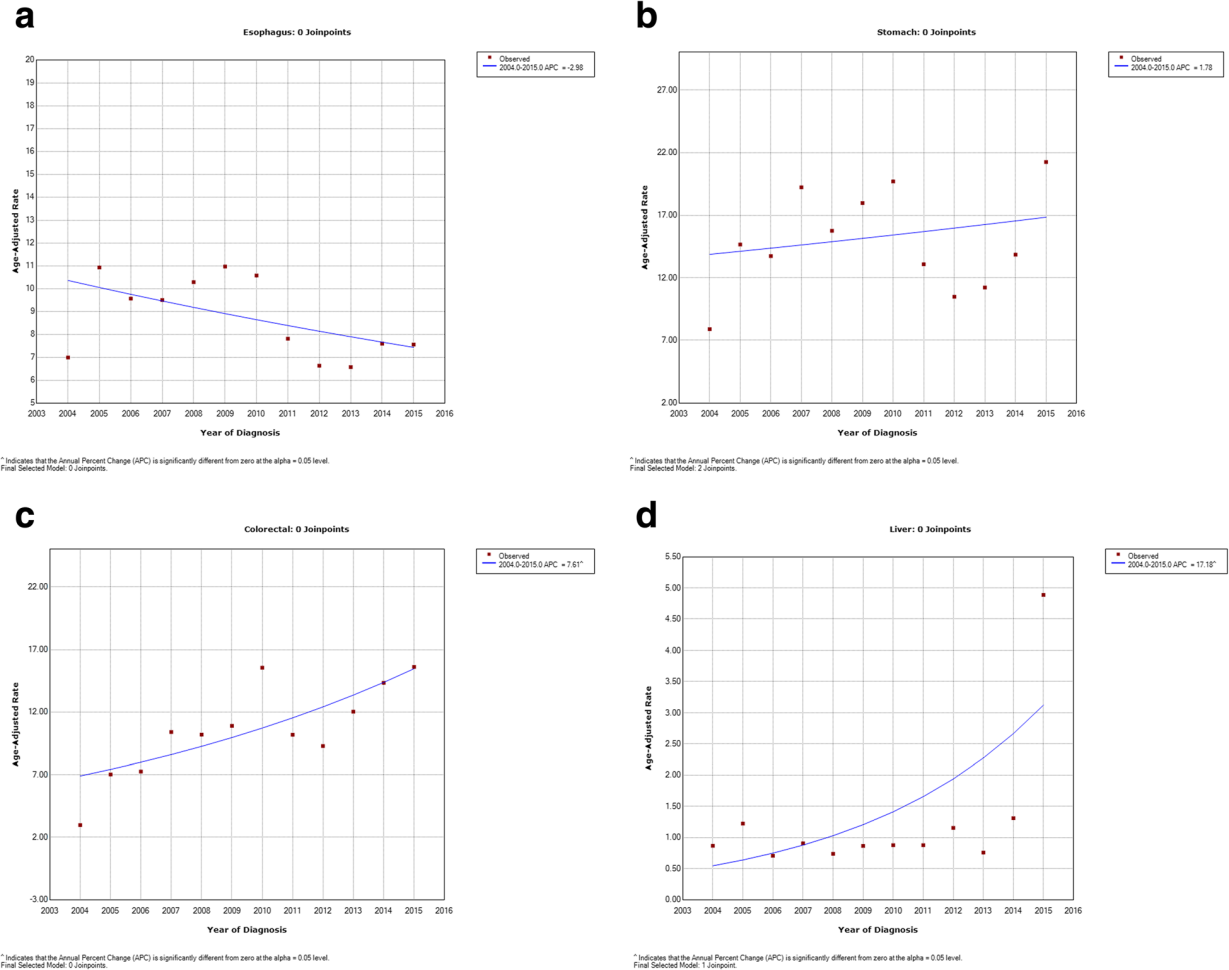
Trend Analysis Results of the Incidence of Gastrointestinal Cancers from the East Azerbaijan Cancer Registry (2004–2015) in Both Sexes: **a**: Esophageal Cancer; **b**: Gastric Cancer; **c**: Colorectal Cancer; **d**: Liver Cancer

**Fig. 2:**
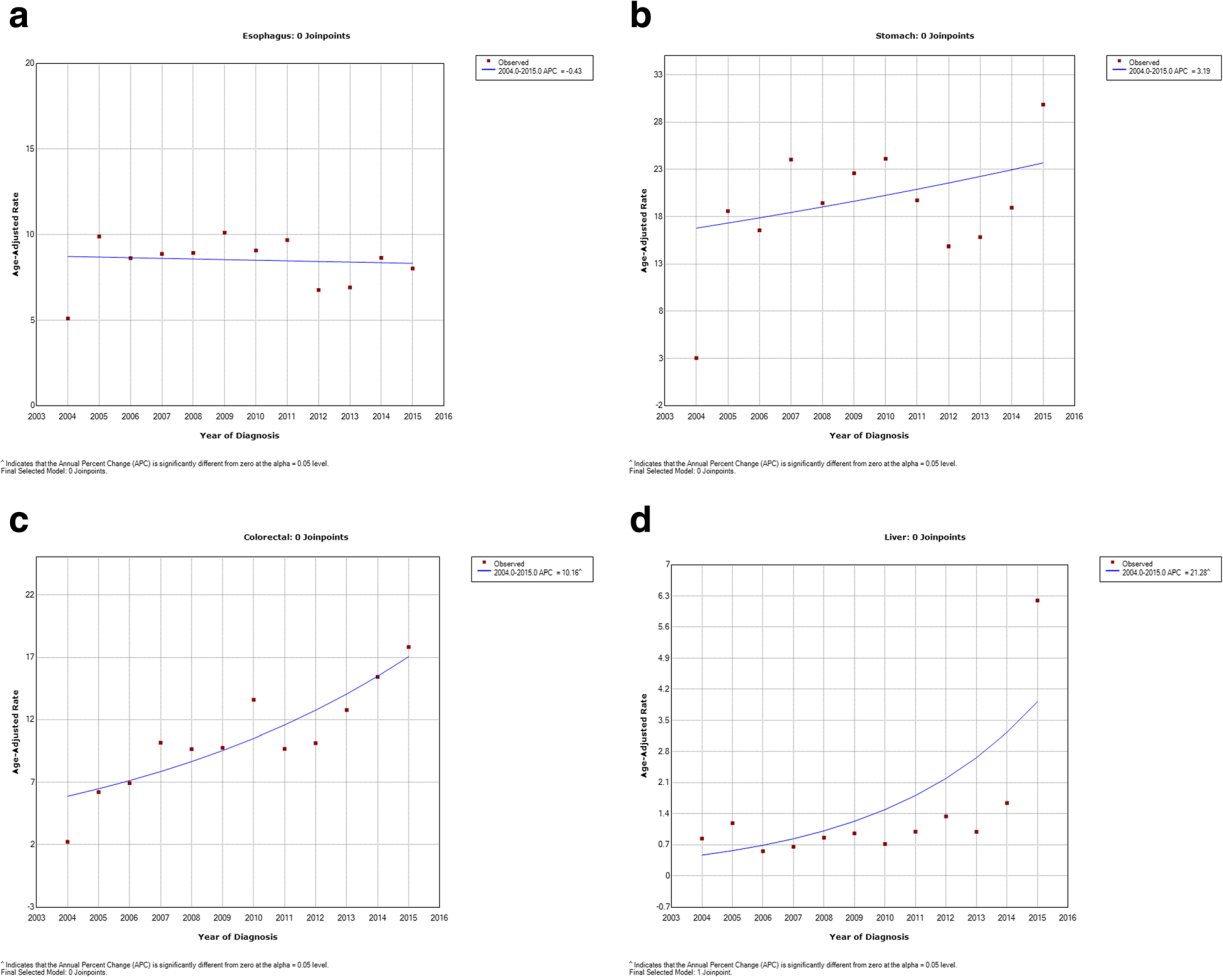
Trend Analysis Results of the Incidence of Gastrointestinal Cancers from the East Azerbaijan Cancer Registry (2004–2015) in Males: **a**: Esophageal Cancer; **b**: Gastric Cancer; **c**: Colorectal Cancer; **d**: Liver Cancer

**Fig. 3:**
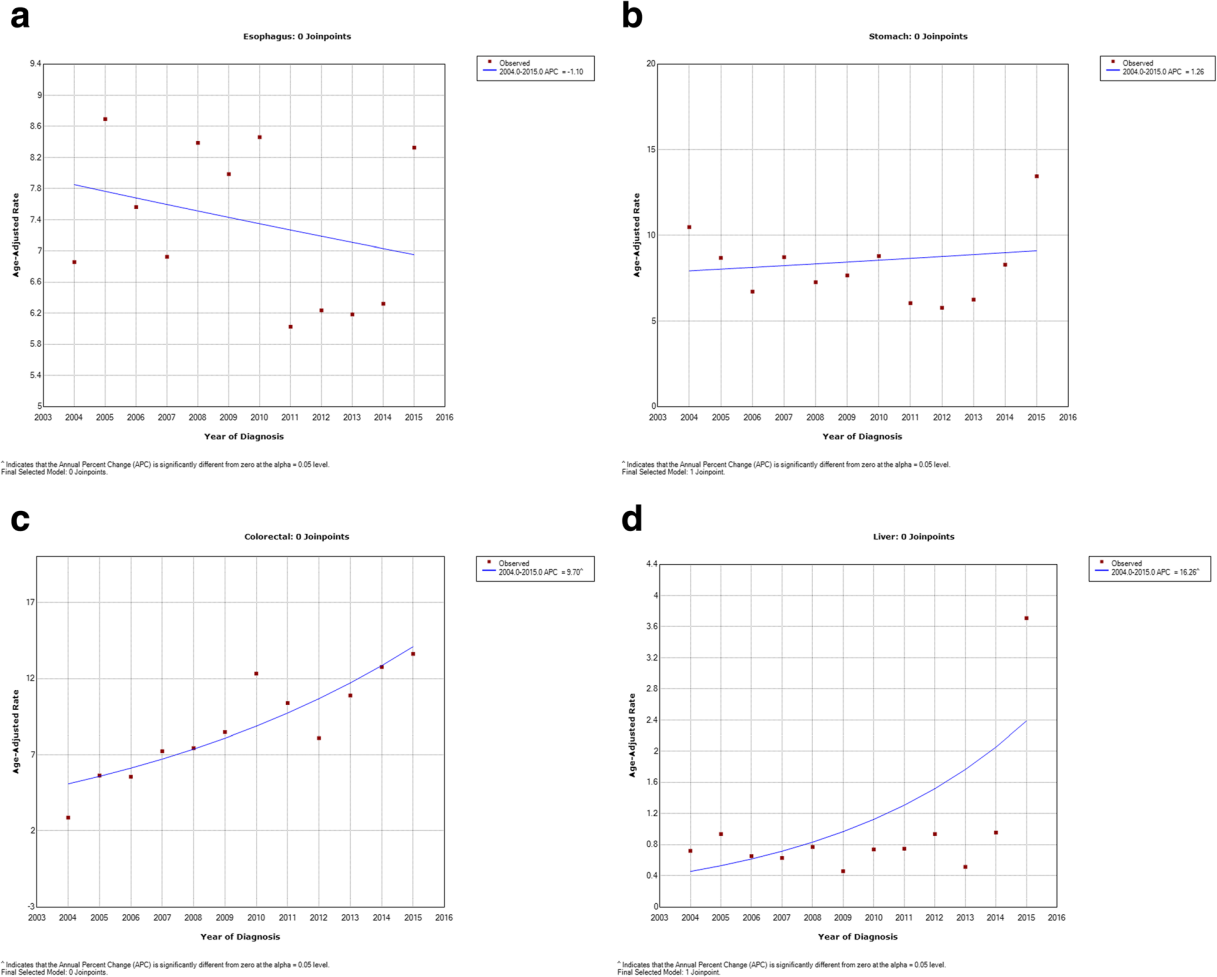
Trend Analysis Results of the Incidence of Gastrointestinal Cancers from the East Azerbaijan Cancer Registry (2004–2015) in Females: **a**: Esophageal Cancer; **b**: Gastric Cancer; **c**: Colorectal Cancer; **d**: Liver Cancer

There was a decreasing trend in the ASRs of esophageal cancer in both sexes, giving an overall APC of − 3% (95% CI: − 6.1 to 0.2). The overall APC in women was − 1.1% (95% CI: − 3.6 to 1.5) compared with − 0.4% (95% CI: − 3.8 to 3.0) in men. The greatest decreases were between the mid Joinpoint in both sexes (2009–2012), with an overall APC of − 16.2%, and APCs of − 8.5% (2009–2013) and − 11.00% (2010–2013) for men and women, respectively (Figs. 1a, 2a, 3a).

### Gastric cancer

There was a trend for gastric cancer to increase in both sexes between 2004 and 2007 (APC, 29.5%) before the ASRs then decreased between 2007 and 2013 (APC, − 9.4%), and finally increased significantly between 2013 and 2015 (APC, 36.1%). The overall APC statistical test for the two Joinpoints was 1.8% in both sexes, was larger in men (APC, 3.2%) than women (APC, 1.3%), and the increasing trends were significant (*P* ≤ 0.05). The increase in gastric cancer ASRs in the first time segment was larger in men. Between the years 2004 and 2007, the APC was 38.1% in men vs. − 11.1% in women between the years 2004 to 2006. Following a slight decreasing trend in both sexes in the second time segment, there was again a significant increase in the trend between 2013 and 2015, with an APC of 34.8% in men and an APC of 45.1% in women (Figs. 1b, 2b, 3b).

### Colorectal cancer

There were non-significant increases in the ASRs for colorectal cancer in both sexes, with an overall APC of 7.6% per year (95% CI: 2.7 to 12.7) (*P* = 0.13) and the highest increases during the first (2004–2009; APC, 23.4%) Joinpoint, following a decreasing trend in the second time segment (2009–2012; APC, − 9.6%), and then increasing in the last Joinpoint (2012–2015; APC, 16. 8%). The increase was slightly lower in women (overall APC, 9.7% per year, 95% CI: 5.7 to 13.8) than in men (10.2% per year, 95% CI: 5.5 to15.1). In women, we observed an increasing trend at the first Joinpoint, with an APC of 18.7% (2004–2010), before seeing a slight decrease (− 3.4%) in the APC between 2010 and 2013, and a further increase of 18.6% in the last 3 years (2013–2015). A slightly different trend was observed in men, with an APC of 46.6% between 2004 and 2007, an APC of 0. 7% between 2007 and 2012, and a final increase of 18.4% between 2012 and 2015 (Figs. 1c, 2c, 3c).

### Liver cancer

Liver cancer showed an overall stable decreasing trend from 2004 to 2013 in both sexes, but in the last three years (2013–2015), there was a high and significant increase in the APC of 138.2% (95% CI: 76.9 to 220.7; *P* ≤ 0.05), prior to which we observed slightly decreasing trends: (2004–2006; APC, − 6.4%) and (2006–2013; APC, − 1.2%). In men, there was a decreasing trend of − 14.5% (95% CI: − 54.6 to 60.8) between 2004 and 2006, then a slightly increasing trend in the mid Joinpoint (2006–2013; APC, 4.4; 95% CI: − 6.4 to 16.5), and a significant increase of 139.9% (95% CI: 62.4 to 254.2; *P* ≤ 0.05) between 2013 and 2015. In contrast, in women we observed a slightly decreasing trend in the two first periods, between 2004 and 2010 APC decreased by − 1.1% (95% CI: − 14.1 to 13.9) and − 6.9% between 2010 and 2013 (95% CI: − 56.8 to 100.7), with a significant increasing APC of 148.0% (95% CI: 45.6 to 322.7; P ≤ 0.05) in the last three years (2013–2015) (Figs. 1d, 2d, 3d).

## Discussion

Our results showed that there was a decreasing trend in the ASRs of esophageal cancer in both sexes, and the greatest decreases were between the mid Joinpoint in both sexes (2009–2012), giving an overall APC of − 3%. There was a significant increasing trend of gastric cancer that was greater in men (APC, 3.2%) than in women (APC, 1.3%). For colorectal cancer there was an increase in the ASRs with an overall APC of 7.6% per year for both sexes. Liver cancer showed an overall stable decreasing trend from 2004 to 2013 in both sexes, but in the last three years of the study (2013–2015) there was a significant increase in the APC of 138.2%.

The first East Azerbaijan Population-Based Cancer Registry was established in Iran in 2001. Although it was originally based on pathology data, it has since been developed and improved to the point where we have established a standardized population-based cancer registry of data obtained from different sources. Cancers of the gastrointestinal tract are among the most common and important cancers in Northwestern Iran and East Azerbaijan [6]. Gastric cancer in particular continues to be the most common among men in this region [4, 6]. Global Cancer Statistics have estimated the incidence and mortality of 36 cancers across 20 world region and in 185 countries, by age and sex. The last updated cancer statistics produced by the International Agency for Research on Cancer (IARC) used reliable cancer incidence and mortality data sources from the various countries [1].

### Gastric cancer

The incidence of gastric cancer is particularly high in Iran, where it remains a leading cause of cancer-related death, and where it is the most common cancer among Iranian men, with an ASR of 21.6 per 100,000 men [1]. The highest ASRs and age-standardized mortality rates (ASMRs) for gastric cancer were reported in northern, particularly northwestern, regions of Iran [11, 12]. The Ardabil province in the northwest, for example, has the highest incidence of gastric cancer, with ASRs of 51.8 (95% CI: 47.8 to 55.8) and 24.9 (95% CI: 21.5 to27.2) per 100,000 men and women, respectively [13–15].

Recent data have shown extremely high incidence and mortality rates for gastric cancer in East Azerbaijan [4, 6, 16], with gastric cancer being the second leading cause of death (10.4% of all deaths) [4]. Other research has shown gastric cancer to have been the most common cancer in northern and northwestern Iran over the past 30 years [11, 14, 17]. It also remains the most common cause of cancer-related death in Iran, albeit with a reported six-fold geographic variation in the mortality rate between northwestern and southern regions [16]. Trend analysis in the current study showed that the incidence of gastric cancer increased in both sexes by an APC of 1.8% per year, but that this was most marked in the last three years (2013–2015), when the APC was 36.1%, and interestingly this was larger in women (APC, 45.1%) than in men (APC, 34.8%). Many studies have shown an increase in the trend for gastric cancer in Iran [13, 18, 19]. Indeed, a significant increase was observed between 2001 and 2010, when the ASRs increased from 4.18 to 17.06 (APC, 16.7%) for men and from 2.41 to 8.85 (APC, 16.2%) for women [20].

Although *Helicobacter pylori* infection is the most common established risk factor for gastric cancer [21–23], many surveys have shown geographic patterns in the incidence and mortality of gastric cancer that correlate with tobacco smoking [20]. Nevertheless, the elevated risk of developing and dying from gastric cancer has been linked to the high prevalence of *H. pylori* infection, tobacco, opium, and specific dietary factors in this region [24–28]. There are also issues surrounding low consumption of fresh vegetables and fruits, low socioeconomic status, high consumption of salt and salty foods, poor lifestyle habits, and certain food preserving methods as risk factors of gastric cancer. Atrophic gastritis is another well-established risk factor, which has also been reported to have a high incidence in parts of Iran [11]. It is notable that, while Iran has faced an increase in the incidence and mortality for gastric cancer, the US and Western Europe have seen declines in both [29], with a decrease of − 20.8% in incidence rates [30], probably because of improved *H. pylori* infection control, the impact of smoking cessation programs, efforts to control salt consumption, and improved dietary habits.

### Colorectal cancer

Colorectal cancer is the third most common cancer and second in terms of mortality from cancer in the world according to GLOBOCAN 2018 [1]. Moreover, the worldwide incidence increased from 1.3 million to 1.7 million cases (overall increase rate of 34%) between 2006 to 2016 years [2]. It has the second highest incidence among cancers in developed countries, and the fourth highest incidence and mortality rate in developing countries [30]. Unfortunately, however, there has been a trend for the incidence of colorectal cancer to increase in countries undergoing developmental transitions, including Iran [1]. According to GLOBOCAN 2018, it is now the third most common cancer in men and the second most common cancer in women [1], though the highest rates have been reported in Tehran (ASR = 16.4 for men) [31] and Zahedan (ASR = 13.3 for women) [32, 33].

Despite these increases, Iran still has a low incidence of colorectal cancer compared with western countries. However, while the incidence and mortality rates of colorectal cancer have decreased steadily in North America and most developed countries, falling by 30% in the last decade, there has been a trend for significant annual increases in the incidence and mortality of colorectal cancer in Iran [19, 33, 34]. Also, the ASRs and crude rates are known to be lower in southern regions of Iran compared with northern and northwestern regions [33, 35, 36]. We consider that nutritional habits, obesity, and genetic and environmental risk factors contribute to these differences [37–39]. Several reports on the ASRs and crude incidence rates of colorectal cancer in East Azerbaijan have shown a trend for the incidence of colorectal cancer to increase, especially in women [3, 4, 33, 40, 41]. According to our trend analysis, there was a trend for a steady increase in the incidence of colorectal cancer in both sexes, with an overall APC of 7.6% (95% CI: 2.7 to 12.7%), although this was more obvious in men (APC, 10.2%) than in women (APC, 9.7%).

### Liver cancer

Globally, liver cancer has increased from the third to the second leading cause of years of life lost to cancer, now ranking as the fourth leading cause of cancer-related death worldwide in 2018 [1]. In Iran, liver cancer is the 10th most common cancer in terms of incidence and the fifth most common cancer in terms of mortality [2]. Some studies have shown a significant rising trend in the ASRs for liver cancer in this country, though an increase with age has been reported to be more common in men [42, 43]. The trend in the ASRs for hepatocellular carcinoma between 2001 and 2008 in Iran showed an increase from 0.4 to 0.6 per 100,000 [43]. Survey updates on the 5-year incidence and trends of gastrointestinal cancer in East Azerbaijan have shown significant increases in the ASRs for liver cancer in both women (0.6) and men (0.9) [4]. Our results showed an increasing trend in the incidence of liver cancer over 12 years, with an APC of 17.2%, and it was more obvious in men than in women (APC, 21.3% vs. 16.3%). Hepatitis B and C are the main risk factors for liver cancer, so hepatitis B vaccination and hepatitis C treatment (albeit expensive) are the recommended proven options to prevent this cancer. Also, the higher increasing trend of incidence of liver cancer in last two years of the study may be attributed to increased coverage of cancer registry data and including more data sources such as imaging and death registry data, which were not collected in the first years’ results.

### Esophageal cancer

Esophageal cancer has a very low incidence and prevalence in most countries, but in Iran, it is the 8th most common cancer and was the ninth cause of cancer death [1]. The highest ASRs have been reported in the Golestan province, where the incidence of esophageal cancer has historically been very high [20, 44]. Recent data have suggested a significant decrease in the ASRs in the high-risk population of Iran [20, 45, 46], and although we also observed a decrease between 2006 and 2013, mostly in women, the ASRs steadily increased again by the last Joinpoint (2013 to 2015). Previous report from East Azerbaijan also showed a decreasing trend in esophageal cancer incidence during 2004 to 2011, with an APC of − 6.9 and − 2.2 in females and males respectively [4]. Also the incidence rate tended to decreased significantly in this region for both Squamous cell carcinoma (SCC) and adenocarcinoma (AC) morphologic types over time 1992 to 2004 [47]. According to the similar reports from other regions of Iran, this decreasing may be attributed to improvement in life style including hot tea drinking habits, smoking, and improvement in socio-economic status [4, 47]. It is believed that esophageal cancer incidence (and especially SCC type) has been declining worldwide and in high-risk areas in Asia as well. Lifestyle and dietary improvements and economic gains and declining in cigarette smoking are the min attributed factors [1]. Public education, eradication of opium addiction, and nutritional support may decline the mortality and morbidity that results from EC worldwide [48].

Whereas several studies have reported a significant correlation between opium use and esophageal or gastric cancer [24, 28, 49], others have shown an association between using different types of opium and pancreatic cancer. In a survey from Tehran University of Medical Sciences, opium use (odds ratio 1.9; 95% CI, 1.1–3.4) and alcohol consumption (odds ratio 4.2; 95% CI: 1.9 to 9.3) were significantly associated with increased risks of pancreatic cancer [50]. The most abundant opium alkaloids (pyrolysates of morphine) have known mutagenic effects that can cause cancers, including pancreatic cancer. Indeed, six opium pyrolysates have been implicated as mutagens in esophageal cancer [51]. According to the most recent Global Burden of Cancer report, the incidence of pancreatic cancer increased globally by 4.9% for both sexes, with a larger relative increase in developing countries [30]. However associations of these risk factors and some other life style factors including smoking, overweight and obesity, and meat consumption with pancreatic cancer risk remain unclear and further research, particularly of long-term intake, is warranted.

## Conclusion

Iran has faced increasing trends in cancer incidence and mortality over recent decades because of population growth, population aging, and increased exposure to risk factors. This survey and trend analysis of gastrointestinal cancers’ burden at the population level in East Azerbaijan, Iran emphasizes the urgent need for focused prevention and intervention programs. However, some limitations should be considered. Of note, the East Azerbaijan Population-Based Cancer Registry is still in its infancy, and during the study period there have been ongoing efforts to improve the quality and coverage of the data registry. As with cancer registry programs in other centers, the quality and coverage of the registry may, therefore, have changed over time. Indeed, the increasing trends reported for the cancers in this study may be attributable to factors such as population growth, population aging, or increased exposure to known risk factors. Although this is possible, we contend that the increases seen in the most recent study period were valid because the improvements to the cancer registry program were established by that time.

## Data Availability

The datasets analyzed and presented in this study are available from the corresponding authors on reasonable request.

## Abbreviations

AAPC: Average annual percent change
APC: Annual percent change
ASMR: Age-Standardized mortality rate
ASR: Age-Standardized incidence rate
CI: Confidence interval
EA-PBCR: East Azerbaijan Population Based Cancer Registry
ICD-O-3: International Classification of Diseases for Oncology, Third Edition
